# Multiple Risk in Pregnancy- Prenatal Risk Constellations and Mother-Infant Interactions, Parenting Stress, and Child Externalizing and Internalizing Behaviors: A Prospective Longitudinal Cohort Study from Pregnancy to 18 Months Postpartum

**DOI:** 10.1007/s10802-023-01145-x

**Published:** 2023-11-08

**Authors:** Beate Helmikstøl, Vibeke Moe, Lars Smith, Eivor Fredriksen

**Affiliations:** 1 Department of Psychology, Ansgar University College, Fredrik Fransons Vei 4, 4635 Kristiansand, Norway; 2 Department of Psychology, University of Oslo, Forskningsveien 3a, 0373 Oslo, Norway

**Keywords:** Prenatal risk, Latent class analysis, Internalizing problems, Externalizing problems, Parenting stress, Mother-child interactions

## Abstract

Multiple risk is associated with adverse developmental outcomes across domains. However, as risk factors tend to cluster, it is important to investigate formation of risk constellations, and how they relate to child and parental outcomes. By means of latent class analysis patterns of prenatal risk factors were identified, and relations to interactional quality, parenting stress, and child internalizing and externalizing behaviors were investigated. An array of prenatal risk factors was assessed in 1036 Norwegian pregnant women participating in a prospective longitudinal community-based study, Little in Norway. Mother-infant interactions were videotaped and scored with the Early Relational Health Screen (ERHS) at 12 months. The Parenting Stress Index (PSI) and Infant-Toddler Social and Emotional Assessment (ITSEA) were administered at 18 months. First, we analyzed response patterns to prenatal risks to identify number and characteristics of latent classes. Second, we investigated whether latent class membership could predict mother-child interactional quality, parenting stress, and child internalizing and externalizing behavior after the child was born. Results revealed three prenatal risk constellations: broad risk (7.52%), mental health risk (21.62%) and low-risk (70.86%). Membership in the broad risk group predicted lower scores on interactional quality, while membership in the mental health risk group predicted less favorable scores on all outcome measures. Prenatal risks clustered together in specific risk constellations that differentially related to parent, child and interactional outcomes.

It is widely recognized that children born into families characterized by multiple risks evince more adverse outcomes across developmental domains than children who experience little or no such risk (Felitti et al., 1998; Sameroff, 1998). These findings extend to the very early stages of life, comprising prenatal risks such as maternal psychopathology, substance use, stress, and sociodemographic factors; as well as the cumulative risk these factors confer on child outcomes (Davis & Narayan, 2020; Stein et al., 2014; Wallander et al., 2019). While studies of cumulative risk have yielded important insight on accumulated effects of multiple risk factors on child development, they have also been criticized for not differentiating among diverse risk factors, and for lacking specificity of risk-outcome pathways (Evans et al., 2013). Identifying prenatal patterns of risk by integrating multiple risks at multiple levels to assess how such factors cluster into separate risk constellations, comprise an alternative approach, currently underexplored. To address this knowledge gap, we conducted latent class analysis (LCA) using a prospective, population-based Norwegian sample of pregnant women and their children (N = 1,036 families), to (i) identify and characterize distinct prenatal risk patterns, and (ii) predict mother-child interactional quality, parenting stress, and child internalizing and externalizing difficulties 12 and 18 months after birth based on these patterns. This allows for investigation of typical combinations of risk factors during pregnancy, as well as providing more domain-specific knowledge on risk-outcome pathways. A few studies have investigated risk constellations using LCAs on samples of pregnant women, typically identifying 3–5 risk patterns (Hemady et al., 2022; Hendryx et al., 2020; Loomans et al., 2013; Molenaar et al., 2023; Mukherjee et al., 2017; Tian et al., 2018). In community samples this approach usually entails a larger low-risk group and several smaller groups characterized by elevated risk. However, some of these studies have narrowly defined exposure variables, restricting the risk constellations to domains such as life stress (Mukherjee et al., 2017), social mobility (Tian et al., 2018), or adverse childhood experiences (Hemady et al., 2022); others have included a lower number of risk indicators (Loomans et al., 2013). Some studies comprise more broadly measured prenatal vulnerabilities (Hendryx et al., 2020; Molenaar et al., 2023), but restrict related outcomes to pregnancy complications or birth-related outcomes. There is a lack of research on the longer-term outcomes of prenatal risk constellations. Studies applying LCAs (not restricted to the prenatal period) on longer-term child outcomes have identified risk classes based on constellations of maternal mental health symptoms, exposure to adverse childhood experiences, prenatal substance exposure, family stress, and sociodemograhipcs (Campbell et al., 2009; Göbel & Cohrdes, 2021; Scotto Rosato & Baer, 2012; Wang et al., 2022). However, although some of these data collections started early in infancy, none started in pregnancy. We aim to fill this gap by investigating child and parental functioning during infancy and toddlerhood as outcomes of prenatal risk patterns. The underlying mechanisms of associations between prenatal risk and later child development are not fully understood, but various pathways have been proposed. These include the continuation of risk from pregnancy to the postnatal period, fetal programming or epigenetic mechanisms, shared genetic vulnerability, and risk factors affecting parental well-being and behaviors (Babenko et al., 2015; Davis & Narayan, 2020; Davis et al., 2011; Goodman & Gotlib, 1999; Leis et al., 2014; Stein et al., 2014). The burden of multiple risks at multiple levels may also accumulate over time (Evans et al., 2013). Although LCAs cannot provide a causal explanatory pathway from prenatal risk to postnatal functioning, investigating distinct risk constellations may prove useful in furthering the understanding of the complex interplay of risk factors present as early as pregnancy. By including factors commonly addressed in primary health care for pregnant women, we aim to inform targeted prevention and intervention strategies of clinical relevance.

## Prenatal Risks and Child, Parental, and Dyadic Outcomes

When studying early child development and parental functioning in new families, we need to acknowledge that prenatal conditions serve as a starting point for postnatal development and family life, and it is important to consider links to dyadic, parental and child functioning. Dyadic interactional quality is influenced by both parental, child and contextual variables, such as education and socioeconomic status (van Doesum et al., 2007), maternal mental health, including prenatal mental health (Bernard et al., 2018; Hakanen et al., 2019; van Doesum et al., 2007), level of overall stress (Neuhauser, 2018), and child characteristics (Bates et al., 2012). Most studies focus on few risk factors, without consideration for how risks group together, and less is known about prenatal influences. This is also the case for parenting stress, as there are few studies of prenatal precursors. Parenting stress is strain confined to the parenting role, wherein the demands of parenting are perceived to exceed available resources and competence. A wide range of postnatal and contextual conditions are considered sources for such stress (i.e. Barroso et al., 2018; Cassells & Evans, 2017; Raphael et al., 2010), but prenatal influences are less well understood. Prenatal predictors include anxiety and depression in pregnancy (Huizink et al., 2017; Misri et al., 2010), and maternal attachment style (Mazzeschi et al., 2015), but more research in this area is warranted. For internalizing and externalizing behaviors in children, investigating risk present before the baby is born is a growing field of inquiry, and factors such as smoking, alcohol consumption, and dietary patterns in pregnancy, maternal mental and physical health, as well as intimate partner violence have been investigated (Clayborne et al., 2021; Khoury et al., 2018; Kingston et al., 2018; Leis et al., 2014; Moylan et al., 2015; Steenweg-de Graaff et al., 2014; Tien et al., 2020). In addition, a variety of postnatal factors, such as maternal mental health, parenting behaviors, child temperament, family environment, and socioeconomic status play a part as well (Goodman et al., 2011; Leve et al., 2005; Mills et al., 2012). Multiple risks have been associated with co-occuring internalizing and externalizing problems in preschoolers (Edwards & Hans, 2015), but as for all the above mentioned outcome measures, how multiple risks as early as in pregnancy relate to later such behaviors remains less well understood.

## Study Aims

A next step for advancing knowledge in this field of inquiry is to study how multiple prenatal risk factors cluster together in distinct risk constellations- and whether these are differentially associated with key child and parental outcomes during infancy. By analyzing a wide array of prenatal risk factors in a community-based prospective sample of pregnant women in Norway (N = 1,036) using latent class analysis, we aim to (i) identify and characterize distinct patterns of risks among the pregnant women; and (ii) examine whether these risk constellations predict mother-child interactions at 12 months, and parenting stress, child internalizing and externalizing difficulties at 18 months. We hypothesize that risk factors aggregate within specific domains, beyond simple differences in levels, intensity or number of risks, and that any rise in risk status is associated with less favorable outcomes. With the current knowledge base on prenatal risk constellations, further hypotheses on the specific number of classes, characterization of risk patterns, and potentially differential associations with outcomes, are not warranted.

## Method

### Sample

Participants are part of the prospective longitudinal community-based cohort study, Little in Norway (Moe et al., 2019). Pregnant women were recruited during pregnancy through routine check-ups at their local well-baby clinic. Nine clinics, representing all four health regions of Norway, participated. All pregnant women in the catchment area of these clinics were invited to participate, and a response rate at 50,7% was obtained. Non-response was due to both lack of invitation (i.e. only some midwives conveyed invitations) as well as women declining to participate, however, we lack exact numbers on this. Questionnaires were administered in Norwegian or English. Informed consent was obtained from all participants. A total of 1,036 mothers completed the data collection package at enrollment, usually mid pregnancy (Mweek = 23.76, range = 8–34, SD = 4.93). Mother-infant interactional quality (ERHS) was assessed 12 months after birth, and parenting stress (PSI) and internalizing and externalizing child behavior (ITSEA) were reported 18 months after birth (see description below). Mothers` age ranged from 17–43 years (Mage = 30.26, SD = 4.78). Most women had college or university backgrounds (77.1%), and 77.3% were full time workers. About half the sample (54.9%) was first-time mothers, and a slight majority of the children born were boys (52.1%).

All participants filled out the enrollment package containing information used for the indicator variables without any missing data. During the course of pregnancy 29 women dropped out of the study, and eventually 1,017 children were born to the study. There was attrition at 12 months (N = 775) and 18 months (ITSEA: N = 658; PSI: N = 716) after birth. For the current study we use mother-infant interaction videos at 12 months. As 105 of the participating families have father-infant/ partner-infant recordings only, these were excluded from analyses, leaving 670 mother-infant interaction video recordings. Participation at the last data collection point (N = 658) was predicted by level of education (OR = 1.01, 95% CI [1.00, 1.11], *p* = 0.044), minority status (OR = 0.43, 95% CI [0.26, 0.73], *p* = 0.002), cohabitation intent (OR = 0.60, 95% CI [0.38, 0.95], *p* = 0.028), pregnancy-related anxiety (OR = 0.98, 95% CI [0.97, 1.00], *p* = 0.029), medication use (OR = 1.97, 95% CI [1.31, 2.95], *p* = 0.001), tobacco use (OR = 0.55, 95% CI [0.34, 0.88], *p* = 0.012), depressive symptoms (OR = 0.93, 95% CI [0.90, 0.96], *p* = 0.001), and adverse childhood experiences (OR = 0.84, 95% CI [0.77, 0.92], *p* > 0.001). Alcohol tolerance, a history of mental health problems and life stress were unrelated to participation at the last data collection point (*p* > 0.05).

Recruitment and data collection were approved by the Regional Committees for Medical and Health Research Ethics in Norway (REK, [2011/560]).

### Measures

*Prenatal Risk Indicators* were chosen to cover multiple domains, including maternal mental health (depressive symptoms, pregnancy related anxiety, previous psychopathology), substance intake in pregnancy (daily smoking/ snus use,^1^ alcohol tolerance, prescribed medications) sociodemographic factors (education level, ethnic minority status, not planning on co-habiting with partner after birth), and current and previous life circumstances (life stress, unwanted pregnancy, and adverse childhood experiences). Information on these 12 indicator variables were obtained by self-report at enrollment and selected on the basis of being topics commonly addressed in prenatal care- and therefore of particular clinical relevance in terms of prevention, early identification, and intervention. All indicators were dichotomized to indicate risk/ no risk. For the Edinburgh Postnatal Depression Scale (EDPS) the cut-off was set at 10, in accordance with a Norwegian validation study on a community sample (Eberhard-Gran et al., 2001), and in line with Norwegian clinical recommendations. The Life Stress Scale cut-off was set at 17, as this warrants clinical referral according to the PSI manual (Abidin, 1995, p. 12). For TWEAK, the cut-off was set at 2 for problem drinking, in accordance with empirical findings (Russell et al., 1996), and clinical recommendations. For Pregnancy Related Anxiety- Revised (PRAQ-R) (Huizink et al., 2004) there is no agreed-upon cut-off. We set the cut-off at 30, leaving 19, 4% of our sample in the risk category. This may be a conservative estimate (Chandra & Nanjundaswamy, 2020). For Adverse Childhood Experiences (ACE) (Felitti et al., 1998) there is also no validated cut-off score, but as exposure to one ACE significantly increases the chance of exposure to multiple ACEs (Dong et al., 2004), the cut-off was set to > 1. Level of education was reported categorically, and high school or less (≤ 12 years) considered non-optimal. The remaining indicators were measured using singular questions framed within a “yes”/ “no” format. See Table 1 for description.

**Table 1:** Description of the 12 Prenatal Risk Indicators

Measure	Description/ Format	Cut-off Used in This Study
Edinburgh Postnatal Depression Scale (EDPS) (Cox et al., 1987)	Self-report questionnaire, 10 items. Responses given on a 4-point scale. Higher scores indicate higher levels of depressive symptoms within the past 7 days. Sum score.	EPDS ≥ 10 non-optimal (Eberhard-Gran et al., 2001)
Pregnancy Related Anxiety Questionnaire-Revised (Huizink et al., 2004)	Self-report questionnaire, 10 items. Responses given on a 5-point scale. Higher scores indicate higher levels of pregnancy-related anxiety (such as fear of giving birth, concerns with bodily changes and/ or concerns for the unborn child). Sum score.	PRAQ > 30 non-optimal
Adverse Childhood Experiences (ACE) (Felitti et al., 1998)	Self-report questionnaire, 10 items. Responses given in a yes/ no format. Higher scores indicate more categories of adverse childhood experiences, covering various forms of abuse, neglect and household dysfunction from age 0–18.	ACE > 1 non-optimal (Dong et al., 2004)
Tolerance Worry Eye-opener Amnesia C(K)ut-down on drinking (TWEAK) (Russell, 1994)	Self-report questionnaire, 5 items. Higher scores indicate problematic drinking habits (i.e. “Does your spouse (or [do your] parents) ever worry or complain about your drinking?”). Sum score.	TWEAK ≥ 2 non-optimal (Russell et al., 1996)
Parenting Stress Index (PSI), Life Stress Scale (Abidin, 1995)	Self-report questionnaire, counts contextual stressors outside of the parent-child relationship in the past 12 months, i.e. experienced deaths in the family, problems at work, moving etc. Responses given in a dichotomous format and weighted according to severity. Higher scores indicate more stress.	Life Stress ≥ 17 non-optimal (Abidin, 1995, p. 12)
Education	Self-report, four categories: 9 years of basic education / high-school / BA degrees / MA degree or more.	High school or less (≤ 12 years) non-optimal
Ethnic minority status	Single question self-report (yes/ no). (“Do you belong to an ethnic minority group?”)	Dummy coded (0 = risk absent)
Not wanting to have this baby	Single question self-report (yes/no). (“Is your pregnancy wanted?”)	Dummy coded (0 = risk absent)
Not co-habit with partner after birth	Single question self-report (yes/no). (“Will you be living with the child`s father after birth?”)	Dummy coded (0 = risk absent)
Any use of prescribed medication in pregnancy	Single question self-report (yes/no). (“Are you on prescribed medication?”)	Dummy coded (0 = risk absent)
Daily smoking/ snus use in pregnancy	Single question self-report (yes/no). (Have you been smoking/ snusing daily during pregnancy?)	Dummy coded (0 = risk absent)
Previous psychopathology	Single question self-report (yes/ no). Framed within a “yes”/ “no” format. (“Have you ever experienced mental health problems earlier in life?”)	Dummy coded (0 = risk absent)

Snus is a non-combustible tobacco product frequently used in the Nordic countries

*Early Relational Health Screen (ERHS)* (Willis, 2007; Willis, Chavez et al., 2020) is a screening instrument for assessing dyadic interactional quality between parent and infant at 6–24 months of age. ERHS addresses the dyad as a unit, not the child`s nor parent`s individual contribution. Mother-infant interactions were videotaped in a play situation by health care nurses. Interactions of 5 minutes were then scored by trained coders. Twenty percent of these recordings were rated by two different coders to ensure adequate inter-rater reliability. At 12 months, seven dimensions are scored; engagement, enjoyment, responsiveness, pacing, attention, initiation, and imitation. These are each rated on a 3-point scale (2 = clearly observed, 1 = sometimes observed, 0 = not observed) (Willis, Condon et al., 2020). The sum score is used, reflecting an overall measure of dyadic interactional quality. Inter-rater reliability was estimated at 0.76, using weighted Kappa, and internal consistency at 0.72 using Cronbachs alpha (Siqveland et al., 2022). Higher scores indicate better interactional quality. For an in-depth description of how the ERHS was administered in this study, see Siqveland et al. (2022).

*Infant-Toddler Social and Emotional Assessment (ITSEA)* assesses social and emotional functioning in children at 12–36 months of age (Carter et al., 2003). It contains 166 items, and parents rated their children on a 3-point Likert scale (0 = not true/ rarely, 1 = somewhat true/ sometimes, 2 = very true/ often). ITSEA contains four broad domains, of which internalizing and externalizing domains were included as outcome measures in our study. The Internalizing Domain includes scales on depression/ withdrawal, general anxiety, separation distress, and inhibition to novelty. The Externalizing Domain includes scales on activity/ impulsivity, aggression/ defiance, and peer aggression. ITSEA was administered when the children were 18 months old. Cronbach’s alpha was estimated to 0.65 for the internalizing domain, and to 0.76 for the externalizing domain.

*Parenting Stress Index (PSI)* (Abidin, 1995) is a self-report questionnaire for parents, intending to map level of stress in the parenting role. Parenting stress is divided into a child domain and a parent domain, each with separate subscales. The Parent Domain, which in this study is used as an outcome measure, contains 48 items answered on a five-point Likert scale (from “Strongly agree” to “Strongly disagree”), covering the following subscales; competence, social isolation, bonding with the child, health, role restrictions, depression, and partner. Higher scores indicate more parenting stress. Chronbach`s alpha was estimated to 0.92.

### Statistical Analyses

To investigate the first research question, response profiles related to the 12 indicator variables were analyzed by means of latent class analysis. The chosen indicators comprise various types of variables; six binary, three sum scores, two comprise counts of life events, and one categorical (see Table 1). Before deciding how to enter these variables in a latent class /profile analysis, variable distributions were examined. The continuous variables, and especially counts of life events were highly skewed. Latent profile analysis is sensitive to skewed distributions, as the more extreme values might drive class solutions, creating small classes differing only on single indicators with extreme values (Sinha et al., 2021). Testing these indicators in preliminary analysis of our data, confirmed this pattern to emerge. When using categorical variables with several levels, having enough observations in each cell might be a problem. As analysis becomes computational demanding with increasing number of classes, the model might not converge (Sinha et al., 2021). It was therefore decided to use binary variables, using cut-off values to indicate risk. Moreover, several of these scales (including cut-offs) are routinely used in perinatal care in Norway, yielding the benefit of being in line with risk assessments in clinical practice. To identify the optimal number of latent classes, the Akaike information criterion (AIC), Bayesian information criterion (BIC), and the sample size-adjusted Bayesian criterion (SABIC) were evaluated. Further, the Lo-Mendell-Rubin test (LMRT) and the bootstrapped likelihood (BLRT) provided information on how a (K-1)-class model compared to a K-class model (Nylund-Gibson & Choi, 2018). The classes were also manually interpreted based on more substantive criteria and class sizes (avoiding small classes, N > 20), and entropy values were examined. 500 sets of starting values were used in the model estimation, along with 50 final stage optimizations, and 50 initial stage iterations, in accordance with recommendations of Geiser (2013). To analyze the second research question, the LCA three-step procedure for auxiliary variables was carried out. This allows for the adding of distal outcome variables (mother-child interactions, parenting stress, child internalizing and externalizing behavior) to the model as multinomial regressions, after the class structure has been established (Asparouhov & Muthén, 2014; Nylund-Gibson & Choi, 2018). Preliminary analyses revealed no confounding between latent class and child gender. Parity was somewhat higher in the low-risk group (M = 0.66) compared to the broad risk (M = 0.42) and the mental health risk group (M = 0.45).

All analyses were conducted using Mplus 8.

## Results

### Descriptive Statistics

There was considerable variation in the exposure to risk factors among the 12 indicator variables, however, for all indicators, the majority reported no risk, see Table 2. Most prevalent risk factors were Adverse Childhood Experiences (36.58%), alcohol tolerance (24.52%), low education (22.88%), and previous mental health problems (21.72%). Least prevalent risk factors were ethnic minority status (6,08%), no intention to co-habit with partner after birth (3.67%), and “not wanting to have this baby” (3.19%).

**Table 2:** Distribution of Dichotomized Risk Scores

Risk Factor	Risk *N*	No Risk *N*	% Risk
Low education	237	799	22.88%
Ethnic minority	63	973	6.08%
No intention to co-habit after birth	38	998	3.67%
Pregnancy not wanted	33	1003	3.19%
Daily smoking/ snus use in pregnancy	76	960	7.34%
Prescribed medication in pregnancy	145	891	14.00%
Previous mental health problems	225	811	21.72%
Pregnancy-related anxiety (PRAQ-R)	201	835	19.40%
Depressive symptoms (EPDS)	100	936	9.65%
Life Stress Scale (PSI)	99	937	9.56%
Alcohol tolerance (TWEAK)	254	782	24.52%
Adverse Childhood Experiences (ACE)	379	657	36.58%

N = 1036

### Description of the Class Identification and Enumeration Process

To investigate the presence of distinct classes of risk patterns in the sample and determine the number of classes, latent class analyses (LCAs) were conducted. Models ranging from 1 to 6 classes were estimated, and several fit indices were used to evaluate model fit, see Tables 3 and 4. It is quite common for various fit indices not to converge, and none of the indices should be interpreted in isolation (Nylund-Gibson & Choi, 2018). The BIC/SABIC suggested a 3-class solution, and although the 6-class solution had the lowest AIC value, solutions beyond 2 or 3 classes did not substantially improve fit. The BLRT indicated a 4-class solution, while the LMRT favored a 2-class solution. For the entropy, values closer to 1 indicate higher classification quality (Geiser, 2013). However, it has been suggested that an entropy of 0.6 is acceptable for publishable papers (Weller et al., 2020). Of the suggested 2 to 4 classes, the 4 class-solution showed the highest entropy. But the model solution should also undergo substantive evaluation, evaluation of the utility of additional classes, and parsimony considerations (Masyn, 2013). It could be argued that 2 classes do not provide enough information, but that 4 classes provide little extra when compared to 3, as the fourth class primarily differed from the baseline class on education. The BIC pointed to 3 classes, and the AIC suggested 2–3. The BIC may be more accurate than the AIC when sample size is large, and the BLRT may outperform the LMRT when using categorical values (Nylund et al., 2007). Both 3 and 4 classes could be argued based on the BLRT. Taken fit indices, substantive reasoning, and the parsimony principle together, the 3-class solution was selected.

**Table 3:** Summary of Fit Indices for Each Class

Model	AIC	BIC	Sample-Size Adjusted BIC, SABIC	Entropy
1 class	9542.88	9602.20	9564.09	
2 classes	9288.47	9412.05^a^	9332.65	0.613
**3 classes**	**9256.70**	**9444.54**	**9323.84**^a^	**0.658**
4 classes	9238.59	9490.69	9328.71	0.718
5 classes	9230.04	9546.40	9342.13	0.768
6 classes	9226.64^a^	9607.26	9362.70	0.795

Overall best fitting model is indicated in bold. N = 1036

^a^indicating the lowest, most favourable value

**Table 4:** Latent Class Comparisons

Model	*p*-value for LMRT (Adjusted Values)	*p*-value for BLRT (Parametric Bootstrap)
1 class vs 2 classes	0.000	0.000
2 classes vs 3 classes	0.306	0.000
3 classes vs 4 classes	0.587	0.000
4 classes vs 5 classes	0.135	0.050
5 classes vs 6 classes	0.360	0.167

### Description of the Risk Constellations

**Fig. 1:**
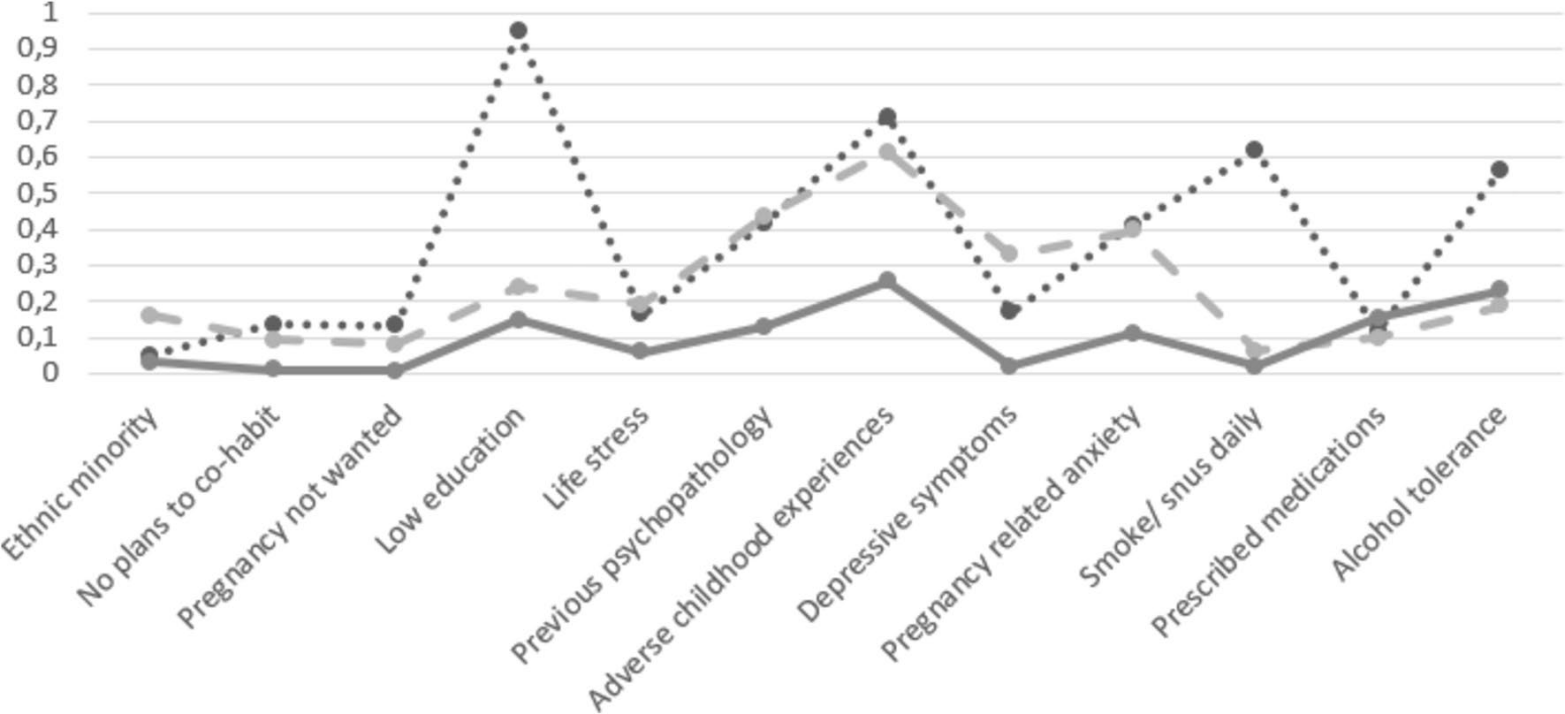
Item Probability for Each Latent Class. *Note*: 12 risk indicators on the x-axis. Item probability on y-axis

Answering the first study aim, we identified three distinct prenatal risk classes among pregnant women by means of latent class analyses, see Fig. 1 and Table 5.

**Table 5:** Conditional Probabilities of Risk by Latent Class

Risk Factor	Class 1 Broad Risk 7.52%	Class 2 Mental Health Risk 21.62%	Class 3 Low Risk 70.86%
Low education	0.947	0.239	0.149
Ethnic minority	0.051	0.160	0.032
Intentions to co-habit	0.135	0.094	0.009
Pregnancy not wanted	0.133	0.079	0.007
Pregnancy related anxiety (PRAQ-R)	0.410	0.395	0.110
Daily smoking/ snus use in pregnancy	0.616	0.063	0.019
Alcohol tolerance in pregnancy (TWEAK)	0.565	0.188	0.229
Life stress (PSI)	0.162	0.191	0.060
Depressive symptoms (EPDS)	0.168	0.328	0.018
Adverse Childhood Experiences (ACE)	0.712	0.613	0.254
Previous mental health problems	0.417	0.437	0.129
On prescribed medication in pregnancy	0.118	0.099	0.155

N = 1036

The *Broad risk class* comprised 7.52% of the sample. This group was characterized by elevated risk probabilities across multiple domains, including lower education (94.7%), adverse childhood experiences (71.2%), daily smoking/ snus use in pregnancy (61.6%), alcohol tolerance (56.5%) and previous mental health problems (41.7%). The *Mental health risk class* covered 21.62% of the sample and comprised an elevated chance of risk exposure primarily associated with mental health vulnerabilities; adverse childhood experiences (61.3%), previous mental health problems (43.7%), pregnancy related anxiety (39.5%), and depressive symptoms in pregnancy (32.8%). The third class included 70.86% of the sample, here termed *Low-risk*. This class was characterized by low risk overall. Most prevalent risk factors were adverse childhood experiences (25.4%), and alcohol tolerance (22.9%).

### Associations Between Risk Constellations and Child and Parental Outcomes

In the final step of the analyses, we investigated if latent class membership could predict quality of mother-child interactions at 12 months, as well as parenting stress, child internalizing and externalizing difficulties at 18 months, see Tables 6 and 7.

**Table 6:** Result of Multinomial Regression. Latent Class Membership Prediction

	Broad Risk vs Low-Risk		Broad Risk vs Mental Health Risk		Low-Risk vs Mental Health Risk	
	Chi square	*p* value	Chi square	*p* value	Chi square	*p* value
Mother-child interactions	7.29	0.007	1.42	0.233	59.63	0.000
Parenting stress	2.46	0.117	6.87	0.009	38.09	0.000
Internalizing behavior	11.50	0.133	1.77	0.183	10.46	0.001
Externalizing behavior	0.26	0.608	5.27	0.022	20.17	0.000

N = 1036

**Table 7:** Mean Scores and Standard Errors for Child and Parental Outcomes

	Broad Risk		Mental Health Risk		Low-Risk	
	Mean	S.E.	Mean	S.E.	Mean	S.E.
Mother-child interactions	9.97	0.68	8.95	0.32	11.84	0.12
Parenting stress	109.60	4.54	126.16	3.33	102.36	1.01
Internalizing behavior	0.47	0.04	0.54	0.03	0.41	0.01
Externalizing behavior	0.45	0.05	0.60	0.03	0.43	0.01

Results for mother-child interactions showed that the broad risk and mental health risk groups scored lower than the low-risk group at 12 months of age, indicating more dyadic interactional difficulties among both risk patterns. There were no differences between the elevated risk groups. On parental outcomes, the mental health group evinced higher scores on parenting stress compared with the two other risk patterns, indicating that the prenatal mental health risk pattern predicted higher levels of parenting stress at 18 months compared with both the low-risk pattern and the broad risk pattern. For child outcomes at 18 months, the mental health risk group was associated with elevated scores on internalizing difficulties relative to the low-risk group; and externalizing relative to both other groups.

## Discussion

The overarching purpose of this study was to investigate the clustering of multiple prenatal risk factors, as well as mapping differential outcomes on key child, parental and interactional outcomes associated with diverse risk constellations. Our first aim was to investigate if distinct subgroups of pregnant women could be identified based on their exposure to a range of prenatal risk factors. Two elevated risk groups and one low-risk group emerged in the data. The smallest group comprising 7.52% of the participants was characterized as a broad risk group. Additionally, a sizable mental health risk group of 21.62% was identified. Finally, the majority of the sample, 70.86%, was characterized by low-risk- as to be expected in a community sample. This highlights how specific risks cluster, providing information on typical risk patterns during pregnancy. It also aligns with previous studies on prenatal risk patterns reporting patterns of sociodemographic, and psychosocial/ mental health risks (Hendryx et al., 2020; Molenaar et al., 2023). In contrast to these studies, we did not find support for additional risk groups in our sample. This may be due to sample and method heterogeneity across studies, or it may reflect that risk patterns clustered around psychosocial stress/ mental health and sociodemographic risk, respectively, constitute more prevalent risk patterns. More studies are needed to confirm these findings.

The *mental health risk* profile represents 21.62% of our sample, underscoring that mental health concerns are common in the perinatal period. This group is characterized by higher probabilities for major depressive symptoms, pregnancy related anxiety, previous mental health problems, and adverse childhood experiences. The clustering of these specific risk factors could reflect that mental health issues overlap in symptomatology and prevalence, and that comorbidity is common (Andreassen et al., 2023; Howard & Khalifeh, 2020). For instance, comorbid depression and anxiety in the perinatal period, may be as high as 20% (Howard & Khalifeh, 2020). This period may also increase risk for a wide range of mental disorders (Howard et al., 2014; Munk-Olsen et al., 2006), and a history of previous psychopathology seems to elevate the risk for a new-onset mental illness (Andersson et al., 2006; Howard et al., 2014). Although the notion that the perinatal period constitutes a special vulnerable period has been debated, mental health challenges are often referred to as the most common complication of childbearing (Howard et al., 2014). Research has also documented clear associations between adverse childhood experiences and later mental health problems (Felitti et al., 1998), in line with the clustering of such experiences into the mental health group in our sample.

*The broad risk* pattern makes up 7.52% of our sample, and is characterized by risk situated across multiple domains, such as socioeconomic factors (lower education), contextual factors (life stress), substance intake during pregnancy (nicotine, snus, alcohol), and mental health factors (adverse childhood experiences, pregnancy related anxiety and previous psychopathology). Still, levels of risk are low in this sample, hence our use of the term “broad risk” as opposed to “high risk”. This group stands out especially in terms of lower education, daily use of nicotine products during pregnancy, and high alcohol tolerance when compared to the other groups. Note that women in the broad risk group also report mental health issues, albeit to a lesser extent depressive symptoms, compared to the mental health risk group. This corresponds with studies showing that several of the risk factors evidenced in this group are commonly reported to co-occur (Evans, 2004; Evans et al., 2013).

### Risk Constellations and Dyadic, Parental, and Child Outcomes

Our second aim was to investigate how these risk constellations related to interactional quality between mother and infant at 12 months, and to parenting stress, child internalizing and externalizing behaviors at 18 months. Aligning with our hypotheses, the mental health group showed less favorable outcomes relative to the low-risk group on all outcome measures. This group also reported more externalizing behavior and parenting stress than the broad risk group. Although it is as expected that multiple risk is associated with more problems for both parents, children, and the dyads, there seems to be some specificity in terms of risk patterns and outcomes. That is, exposure to a narrower set of risk factors, specifically mental health risks, were associated with more problems than exposure to a wider range of risk factors.

Mental health is often reported to constitute a specific kind of risk in the early parent-child interactions, and we expected this to play out in our sample. Across cultures and different SES groups, maternal mental disorders (pre- and postnatally) have repeatedly been found to reduce mothers` ability to sensitively read and respond to infant cues (Anke et al., 2019; Bernard et al., 2018; Dix & Yan, 2014; Field, 2010; Hakanen et al., 2019; Newman et al., 2007). It should be noted that maternal distress in pregnancy also predicts negative emotionality in their babies (Field, 2017; Kling et al., 2023), possibly making interactions more difficult to manage. Our findings add to this literature, as dyads in the mental health group showed significantly poorer interactional quality than dyads with little or no prenatal risk. The women in the mental health group further reported higher stress scores than participants in the two other groups. In the literature, mental health issues in pregnancy and adverse childhood experiences have been linked to later parenting stress (Huizink et al., 2017; Lange et al., 2019). This may be due to mental health issues hindering proper parenting preparations, reducing maternal self-efficacy (Wernand et al., 2014) and/ or impacting stress and behavioral responses in the infant (Huizink et al., 2017). These processes act bi-directionally (Doiron & Stack, 2017), wherein the mother struggles to soothe the child, thereby increasing difficult behavior in the child, further elevating parenting stress. This may be especially relevant in a longitudinal context. The finding of elevated internalizing and externalizing difficulties in the children at 18 months was as expected for a pattern of prenatal mental health risks, as maternal prenatal psychopathology repeatedly has been linked to child social, emotional, and behavioral maladjustment (Clayborne et al., 2021; Davis et al., 2007). Various explanations have been suggested to account for this association. One line of argument relates to genetics and/ or neurobiological development in utero. Although our study does not include data suited to uncover genetic or epigenetic mechanisms, it is plausible that such mechanisms are effective. Prenatal distress may alter epigenetic regulation in utero, placing the baby at elevated risk for future maladjustment (Babenko et al., 2015). Furthermore, the concept of shared genetic vulnerability applies both to same category mental disorders and across categories (Andreassen et al., 2023). With the mental health risk group in our study encompassing several mental health risk indicators, this may render these dyads especially vulnerable through shared genetics. Another proposed mechanism relates to how mental health issues affect parenting through reduced sensitivity in interactions, with mothers failing to respond adequately and regulate the child properly, or even displaying more overtly negative or intrusive parenting (Choe et al., 2013; Clayborne et al., 2021; Dix & Yan, 2014)- all of which may elicit internalizing or externalizing responses in the child, but also elicit parenting stress and affect daily interactions. A recent meta-analytic review found that parental sensitivity was related to both types of behaviors, but with a stronger association for externalizing than for internalizing (Cooke et al., 2022), which concurs with our findings. It may be that maternal mental health plays a larger part in the development of externalizing behaviors. Aversive child behaviors have been found to elicit negative parenting responses in highly depressed mothers- further increasing maladjustment (Dix & Yan, 2014). Such behaviors might be seen as a reaction to maternal insensitivity or to the lack of responsivity frequently associated with maternal psychopathology, as discussed above. Curiously, there is a dearth of literature explaining how comorbid conditions may affect interactions, parenting stress and child behaviors. This is striking, as various mental health risks tend to cluster together, as shown in our study.

The broad risk group was the only constellation that evinced elevated risk probabilities across multiple domains. Along with the mental health group, this group displayed significantly poorer interactional quality than the low-risk group. Still, we speculate that underlying mechanisms for the two elevated risk groups may differ. Previous findings point to mothers` sensitivity in interactions being reduced when overall stress is elevated (Neuhauser, 2018). Furthermore, high SES parents tend to display more responsive and sensitive parenting behaviors in interactions (Paulussen-Hoogeboom et al., 2007; Piccinini et al., 2010). Higher levels of education/ SES have been suggested to reflect educated mothers reading up on child development and having more cognitive resources available to adjust parenting strategies (Bornstein et al., 2010). Perhaps it is not SES per se, but rather the cumulative effect of the many co-occurring risk factors of low SES, such as more instability, less social support, and lower quality services, that lead to the accumulation of negative outcomes (Evans, 2004). However, the broad risk group also overlaps to some degree with the mental health risk group in terms of risk exposure. For instance, life stress, pregnancy related anxiety, previous psychopathology, and adverse childhood experiences are almost equally distributed in the two elevated risk groups. These factors may all contribute to difficulties that play out for both risk groups in the dyadic interactions. The combination of mental health risks with these other types of risks may particularly affect maternal reactivity and sensitivity (Mertesacker et al., 2004). Contrary to our hypotheses, we did not find significant differences in parenting stress, internalizing and externalizing behaviors between the broad risk and the low-risk group, although previous research has shown associations between parenting stress and sociodemographic factors, such as ethnicity and socioeconomic status (Cassells & Evans, 2017; Raphael et al., 2010). Worldwide mental health challenges are heavily associated with social inequality (World Health Organization, 2013). When the social risk factors, do not stand out more in our study, it may reflect that mean scores for each risk indicator are low, even within the broad risk group. It could also reflect access to free health care and perinatal follow-up and generous social policies of paid parental leave in Norway, potentially mitigating some of the burden of sociodemographic risk on child and parental outcomes reported above. Consequently, this may play out differently in countries where health care is less accessible and/ or costly. A recent meta-analytic review found stronger associations between internalizing and parental sensitivity in studies with low SES-samples (Cooke et al., 2022), rendering it open to speculation whether the low proportion of low-SES participants in our sample may bias results. Because the narrower mental health group had scores higher on parenting stress and problematic child behaviors, one might wonder whether ongoing (rather than previous) psychopathology, or even depression specifically (which is much more common in the mental health group) contributes more to these difficulties. One might further speculate that the mental health challenges that exist without additional stressors, may be less reactive, and perhaps to a greater extent reflect genetic risk- a genetic vulnerability shared with the child. For the broad risk group, mental health issues may to a lesser extent be associated with genetic vulnerabilities, but rather understood in relation to the total burden of contextual stressors. If this is the case, then broad risk exposure will have more detrimental consequences in contexts without established social welfare systems. Still, a strong social safety net may to a lesser extent alleviate consequences of severe, ongoing psychopathology.

## Limitations

As with all longitudinal studies, attrition is an issue. Attrition in our study was to some extent related to social and demographic diversity. Although not uncommon in psychological research, it remains a limitation that may limit generalizability. There were no missing data for the indicators used to estimate the latent classes, but missingness in outcome variables does warrant some caution in interpretation of these results. Results could be biased towards representing healthy and resourceful women.

Second, although common in community populations, our sample evinced little risk. Even within the broad-risk group, levels of reported risk were relatively low. Few participants were of ethnic minority (perhaps due to language requirements for participation) or single parents (perhaps due to data collection starting relatively early in pregnancy). Also, level of education at the sites of data collection was somewhat higher for participants in this study than for the population in general (Moe et al., 2019). It is unclear how our results translate to a more diverse or high-risk population, and research on different samples and contexts with other distributions of risk is warranted.

Several of the risk factors were dichotomized according to cut-off scores, offering no information on intensity nor duration. Although common practice in cumulative risk research, this is evidently a limitation (Evans et al., 2013). Validity and reliability of the measures vary, and for PRAQ-R and ACE there are no validated cut-offs. Our indicators are not a complete overview of all potential risks, and exposure to protective factors may be just as important for child development.

Within each latent class there is considerable variance. Even in high-risk populations, outcomes are usually largely heterogeneous (Lanza & Cooper, 2016). Further, in naming the classes, there is always a possibility of committing the “naming fallacy”, when complex data is to be labeled by the researcher. This remains a limitation of LCAs (Weller et al., 2020).

Mental health has been highlighted as a specific kind of risk in this study. There is of course the possibility that psychopathology colors mothers` perception of their children, causing them to report their children as more symptomatic (Wesselhoeft et al., 2021). However, this does not apply to interactional quality, which was assessed by means of observation.

Finally, this study investigated mothers only. Yet, characteristics of the other parent may either increase risk or buffer against risk associated with one parent (Martin et al., 2022).

## Clinical Implications

This study provides insights into patterns of risk experienced by pregnant women, and their association with salient parent, child and interactional outcomes in early childhood. Results may aid health care professionals in identification of risk during pregnancy, and in tailoring early and targeted interventions to promote maternal and infant mental health. Based on the findings, health care service providers should be particularly mindful of women with mental health challenges, as these tend to cluster, and pose elevated risk for child and parental functioning. Thus, narrower defined mental health risks may represent an even bigger risk in new families than exposure to a broader set of risks- at least in contexts with strong social safety nets. While access to public health care and social services may partly buffer against sociodemographic and contextual risks, it may not equally buffer against the impact of maternal mental health problems on interactions, parenting stress, and child development. If results prove robust across replications, further exploration on pathways and mechanisms is encouraged.

## Data Availability

Data can be made available upon reasonable request.

## Footnotes

1 Snus is a non-combustible tobacco product frequently used in the Nordic countries.
